# Pott's Disease in a 2-Year-Old Child Treated by Decompression and Anterior-Posterior Instrumented Fusion

**DOI:** 10.1155/2014/252973

**Published:** 2014-03-11

**Authors:** Mehmet Nuri Erdem, Cem Sever, Mehmet Fatih Korkmaz, Sinan Karaca, Ferit Kirac, Mehmet Tezer

**Affiliations:** ^1^Department of Orthopaedic Surgery, International Kolan Hospital, Darülaceza Caddesi No. 14 Okmeydanı ,34360 Istanbul, Turkey; ^2^Department of Orthopaedic Surgery, School of Medicine, Mevlana University, Dökümcü Sk No. 7, Meram Merkez, 42090 Konya, Turkey; ^3^Department of Orthopaedic Surgery, School of Medicine, Inonu University, Malatya Merkez, 44000 Malatya, Turkey; ^4^Department of Orthopaedic Surgery, Fatih Sultan Mehmet Training and Research Hospital Atasehir, 34758 Istanbul, Turkey; ^5^Department of Orthopaedic Surgery, Diyarbakır Training and Research Hospital, Ückuyular Mevki TOKİ karşısı, 21010 Diyarbakır, Turkey

## Abstract

*Introduction*. Paraplegia and kyphotic deformity are two major disease-related problems of spinal tuberculosis, especially in the early age disease. In this study a 2-year-old boy who underwent surgical decompression, correction, and 360° instrumented fusion via simultaneous anterior-posterior technique for Pott's disease was reported. *Case Report*. A 2-year-and-9-month-old boy presented with severe back pain and paraparesis of one-month duration. Thoracic magnetic resonance imaging demonstrated destruction with a large paraspinal abscess involving T5-T6-T7 levels, compressing the spinal cord. The paraspinal abscess drained and three-level corpectomy was performed at T5-6-7 with transthoracic approach. Anterior instrumentation and fusion was performed with structural 1 autogenous fibula and rib graft using screw-rod system. In prone position pedicle screws were inserted at T4 and T8 levels and rods were placed. Six months after surgery, there was no weakness or paraparesis and no correction loss at the end of follow-up period. *Discussion*. In cases of vertebral osteomyelitis with severe anterior column destruction in the very early child ages the use of anterior structural grafts and instrumentation in combination with posterior instrumentation is safe and effective in maintenance of the correction achieved and allows efficient stabilization and early mobilization.

## 1. Introduction

Spinal tuberculosis, the most common pattern of extra pulmonary tuberculosis, also has increased rapidly around the world in recent years, especially in the undeveloped and developing countries [1]. Paraplegia and kyphotic deformity development are two major disease-related problems. Medical and surgical decompression (antituberculous treatment and debridement) and early reconstruction of spinal stability plays an important role in the surgical management of spinal tuberculosis [2, 3]. The reported techniques of surgical treatment range from anterior debridement and interbody fusion [4, 5] and debridement and internal fixation from the posterior approach [6] to combined single-stage or two-stage posterior instrumentation with anterior debridement and bone grafting [7–14]. Recently decompression, fusion, and instrumentation by simultaneous posterior-anterior-posterior surgery are recommended for effective kyphosis correction and early ambulation [15]. In this study a 2-year-old boy who suffered from severe paraparesia and back pain was reported. The aim of this case report is to verify the importance of early reconstruction of spinal stability and the technique of 360° fusion via simultaneous anterior-posterior in a 2-year-old patient.

## 2. Case Presentation

**Figure 1:**
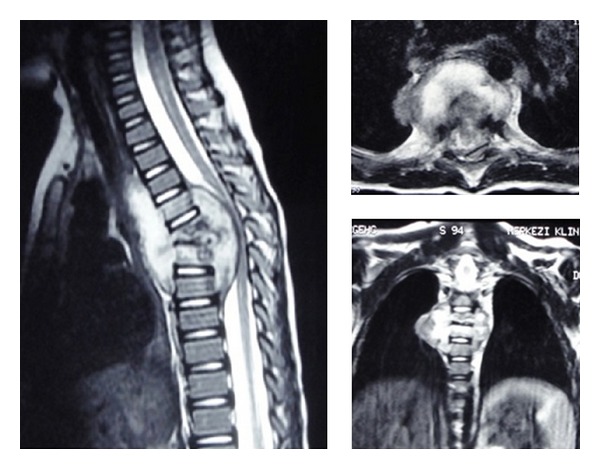
Preoperative MR images demonstrated a large paraspinal abscess.

A 2-year-and-9-month-old boy was admitted to our department with back pain that had developed over a 1-month period. Neurological examination revealed a paraparesis, which was evaluated as grade C according to Frankel Scoring System. Roentgenograms of chest and thoracic spine showed destruction at the T5-T6-T7 levels and the local kyphosis angle was 37° at this level. Thoracic magnetic resonance imaging (MRI) demonstrated a large paraspinal abscess involving these three levels, extending to the spinal canal and compressing the spinal cord (Figure 1). There was no other infection focus in physical examination, radiological, hematological, and biochemical tests. Emerge surgical procedure was performed after preoperative evaluation was completed.

**Figure 2:**
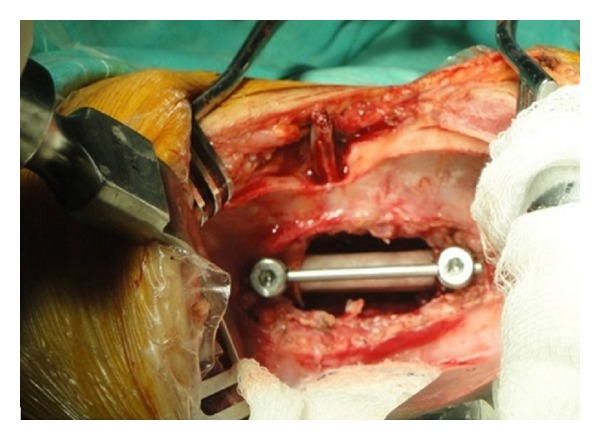
Anterior instrumentation with structural autogenous fibula and autogenous rib graft.

**Figure 3:**
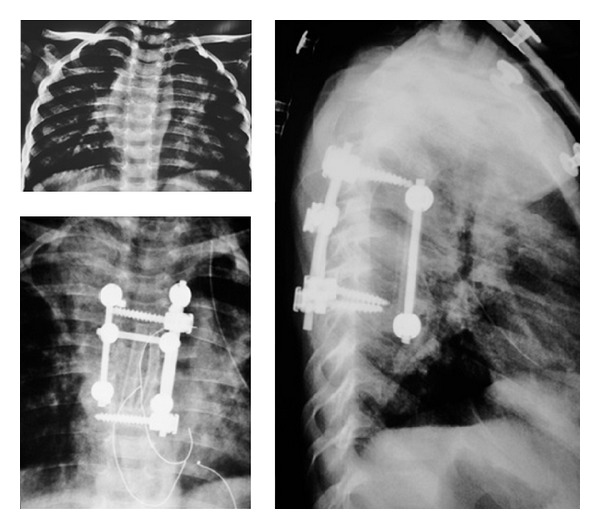
Preoperative and postoperative plain roentgenograms.

The surgery was performed under general anesthesia with the transthoracic approach after removing the fifth rib in right lateral decubit position. After exposure, the paraspinal abscess drained spontaneously. Because of the bone destruction and cord compression, three-level corpectomy was performed at T5-6-7. Sequestered bone fragments of affected vertebrae were removed through corpectomy. Decompression was carried out until duramater was visible through anterior approach, so all affected vertebrae were extracted. The deformity was flexible; the reduction of kyphosis performed until physiological angels and anterior fusion was performed with structural 1 autogenous fibula and 1 autogenous rib graft. Periost of the fibula was prevented and closed anatomically after resection. Anterior instrumentation with one screw to each vertebra at T4 and T8 was performed to increase the primer stability (Figure 2). The system locked, thorax drainage system applied, and thoracotomy closed. The patient was positioned prone. Bilateral polyaxial pedicle screws were inserted at T4 and T8 levels and rods were placed for permanent fixation (Figure 3). Pediatric spinal screw systems were preferred for the immature pedicles (Tasarım med, Istanbul, Turkey).

Microbiological examination of tissue and abscess samples obtained during operation was Mycobacterium Tuberculosis. No complication of bleeding persisted and the drainage tube was taken after 72 hours. One week after surgery, the patient could sit on the bed or walk around under the effective support of thoracolumbosacral orthoses, and this orthoses support was maintained for 3 months. The patient received antituberculosis chemotherapy for 9 months (3 months of INH-rifampicin-pyrazinamide-streptomycin therapy, followed by 6-month INH-rifampicin-pyrazinamide therapy). 6 weeks after surgery, neurological status of the patient was Frankel E and there was no weakness or paresthesia which was presented before the surgery. Kyphosis angle showed a correction of a 4° (89.2%) in the postoperative period and there was no correction loss at the end of follow-up period.

## 3. Discussion

Tuberculosis of the spine is an unstable lesion that tends to progress until having bony fusion at the spine. Bone fusion of the vertebrae is thought to be the most reliable evidence of healing of spinal tuberculosis [16]. To achieve this aim, many treatment methods have been developed, chemotherapy alone and chemotherapy combined with surgical management. Indications for surgery in spinal tuberculosis are reported to include the presence of a large paraspinal abscess, the presence of severe bone destruction and kyphotic deformity, neurologic deficit with spinal cord compression, and lack of response to conservative treatment [17].

The aims of spinal tuberculosis treatment are to eradicate the disease, to prevent the development of paraplegia and kyphotic deformity, to manage the existing deformity and neurological deficit, to allow early ambulation, and to return the patient back to daily life [1, 18–21].

There are many options for surgical treatment of spinal tuberculosis including anterior debridement and arthrodesis [22, 23], plates and screws [3, 5, 24], titanium mesh cages [10, 25], and posterior instrumentation combined with anterior decompression in single or consecutive two stages [15, 26–30]. Anterior debridement and fusion using autogenous bone grafts, as a classical method, allow effective debridement and rapid bony union with the grafts and prevent progressive collapse and kyphosis [22, 31–33]. However, it has been reported that such successful outcomes are not observed regarding progressive collapse and kyphosis, particularly in the case of two or more levels of involvement, risks of graft insufficiency, and increased kyphotic deformity [1, 5, 18, 19, 34–36]. The reported drawbacks of anterior radical surgery suggest that anterior arthrodesis combined with anterior or posterior instrumentation may be useful to maintain the correction achieved [5, 8, 24, 29, 35, 37]. In our experience 360° spinal fusion method using posterior-anterior-posterior titanium mesh cage and posterior instrumentation in tuberculosis spondylitis affecting two or more levels accompanied by moderate or severe kyphosis that requires surgery achieves kyphosis correction comparable to normal anatomic degrees, allows efficient stabilization and early mobilization, and is effective in maintenance of the correction achieved [15]. In this case we prefer to start with anterior debridement and instrumentation because of the flexibility of the deformity due to his age.

In the literature, there are few reports about surgical treatment of spinal tuberculosis in early child ages. To our knowledge this is the first report that shows the surgical treatment which is applied with anterior debridement, anterior instrumentation with structural autogenously fibula, and rib graft and posterior transpedicular instrumentation in early childhood.

In conclusion, in cases of vertebral osteomyelitis with severe anterior column destruction in the early child ages the use of anterior structural grafts and instrumentation in combination with posterior instrumentation is safe and effective in maintenance of the correction achieved and allows efficient stabilization and early mobilization. Further follow-up and series with more account of patients are necessary to confirm these early results.
